# Determination of the influence of weather and air constituents on aortic aneurysm ruptures

**DOI:** 10.1016/j.heliyon.2022.e09263

**Published:** 2022-04-09

**Authors:** Irena Kaspar-Ott, Patrick Olschewski, Stephanie Koller, Alexander Hyhlik-Duerr, Elena Streck, Hans-Henning Eckstein, Oksana Radu, Elke Hertig

**Affiliations:** a Regional Climate Change and Health, Faculty of Medicine, University of Augsburg, Universitätsstraße 2, 86159 Augsburg, Germany; b Clinic for Vascular and Endovascular Surgery, Augsburg University Hospital, Stenglinstraße 2, 86156 Augsburg, Germany; c Department for Vascular Surgery, Klinikum rechts der Isar, Technical University of Munich, Ismaningerstraße 22, 81675 München, Germany

**Keywords:** Aortic aneurysm ruptures, rAA, Germany, Weather, Airborne substances, Atmospheric circulation

## Abstract

In this article, we present a method to determine the influence of meteorology and air pollutants on ruptured aortic aneurysm (rAA). In contrast to previous studies, our work takes into account highly resolved seasonal relationships, a time-lagged effect relationship of up to two weeks, and furthermore, potential confounding influences between the meteorological and air-hygienic variables are considered and eliminated using a cross-over procedure. We demonstrate the application of the method using the cities of Augsburg and Munich in southern Germany as examples, where a total of 152 rAA can be analyzed for the years 2010–2019. With the help of a Wilcoxon rank-sum test and the analysis of the atmospheric circulation, typical weather situations could be identified that have an influence on the occurrence of rAA in the southern German region. These are a rainy northwest wind-type in spring, humid weather in summer and warm southwest wind-type weather in autumn and winter.

## Highlights

• Influence of meteorology and air pollutants on ruptured aortic aneurysm (rAA).
• Consideration of time lags within flexible high temporal resolution analyses.
• Case-crossover procedure ensures that only relevant variables influence the results.
• Developed methodology can be applied to all regions of the world.
• In southern Germany, specific weather conditions significantly influence rAA.

## Graphical abstract

**Figure:**
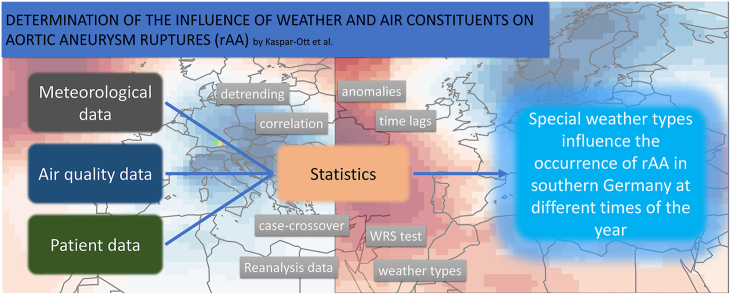

## 1 Introduction

An aortic aneurysm is a spindle- or sac-shaped enlargement of the aorta that can occur in any area of the aorta. The aneurysm is at risk of rupture once the aortic wall stress reaches a certain extent, which could be a fatal threat. Aortic aneurysm most commonly affects individuals over the age of 65, with men developing the aneurysm approximately five times more often than women. The risk of aortic aneurysm increases with age. The reason is that the structure of the vessel wall changes over the years. It is less elastic and can no longer absorb the high pressure which is increasing with diameter enlargement of the aorta (Sidloff et al., 2014).

In more than 50 percent of cases, atherosclerosis (vascular calcification) is the cause of an aortic aneurysm. It also frequently develops in people with high blood pressure (hypertension). High blood pressure puts strain on the vessels and is also a risk factor for atherosclerosis.

The risks for life-threatening rupture of the aorta are primarily hypertension, smoking, and male sex (Hatakeyama et al., 2001). Although the prevalence of aortic aneurysms is much lower in women, the risk of rupture is three times higher than in men with an aortic diameter of 5–6 cm (Heider et al., 2007). Several studies have also investigated other potential influences, such as a dependence on seasons or meteorological conditions. However, these studies show a very high heterogeneity regarding their results (Choong et al., 2019). Most frequently, the influence of atmospheric pressure and air temperature on aortic ruptures was investigated, and individual studies also examined the influence of sunshine duration or lunar phases (Ishikawa et al., 2012; Kózka et al., 2014). In many publications, no influence of weather conditions on ruptured aortic aneurysms (rAA) could be found at all.

Because of the heterogeneity of the results obtained in recent years and the sometimes immature statistical methods that have been used, we have developed a statistical approach that takes into account as many potential sources of error as possible. In doing so, we do not limit ourselves to the meteorological variables mainly mentioned in the literature, such as air pressure and temperature, but extend the spectrum of investigation to include other important variables such as air humidity, precipitation, wind speed and wind direction. In addition, the influence of air pollutants such as ozone and particulate matter are also investigated. For this purpose, in addition to meteorological and air-hygienic measurement data, we retrospectively investigated patient data on rAA from two university hospitals in southern Germany (from the cities of Augsburg and Munich) for a ten-year period (2010–2019).

Our results are based primarily on the use of Wilcoxon rank-sum tests (WRS) to distinguish meteorological and air-hygienic conditions on days followed by rAA from days not followed by aortic ruptures. In contrast to previous studies, our work takes into account highly resolved seasonal relationships with continuous, shifting 3-month analyses to find seasonal differences in the aortic rupture-weather relationship. A fixed division into annual periods (winter, summer, or DJF or comparable) is avoided, because these have no causality in the aortic rupture-weather relationship. The addition of so-called lag days (i.e., the days preceding the rupture) allows, on the one hand, the investigation of time-lagged associations between weather events and aortic ruptures and, on the other hand, the sample can be enlarged in this way, leading to more robust statistical conclusions. Furthermore, potential influences of meteorological and air-hygienic variables on each other are considered and eliminated using a case-crossover procedure in advance of the WKS tests.

Then, all variables of the significant results of the WRS tests occurring at the same time of year are considered together and are used to explain the results of the WRS tests from a climatological point of view. In this way, we identify seasonally different weather conditions that could have an influence on the occurrence of rAA in southern Germany.

Section 2 describes the data and the methodological approach of our study while section 3 again highlights in more detail the results of previous studies on rAA and a potential meteorological influence. The results of our analyses are explained in section 4, which are discussed in section 5. Section 6 briefly summarizes our research findings.

## 2 Data and methods

### 2.1 Data

To be able to investigate potential influences of weather or air pollution on rAA the following data were used: Patient data with the date of rAA and indication of place of residence by postal codes from the University Hospital Augsburg and the University Hospital rechts der Isar in Munich from the years 2010–2019. Data include ruptures of abdominal aortic aneurysms (AAA), thoracoabdominal aortic aneurysms (TAAA) and thoracic aortic aneurysms (TAA), with the former accounting for by far the largest proportion.

Daily weather data from the German Weather Service (Deutscher Wetterdienst, DWD) for air temperature (minimum: tmin, maximum: tmax and daily mean: tmean), atmospheric air pressure (slp), relative humidity (rhum), precipitation (prec), wind speed (windv), and wind direction (windd) for 2010–2019 at the DWD stations Augsburg-Mühlhausen and Munich-City (DWD Climate Data Center, 2020).

Daily data on air constituents from the Bavarian State Office for the Environment (LfU), for ozone (O_3_), nitrogen monoxide (NO), nitrogen dioxide (NO_2_), PM2.5 (particulate matter, size <2.5 μm), and PM10 (particulate matter, size <10 μm) as daily means and daily maxima for the monitoring stations Augsburg/LfU (suburban background), Munich/Lothstraße (urban background), and Munich/Johanneskirchen (suburban background) for 2010–2019 (Bayerisches Landesamt für Umwelt, 2020).

For circulation-based analyses daily mean values of sea level pressure (slp) from the ERA5 reanalysis (Hersbach et al., 2018) for the years 2010–2019 are used. They were interpolated to 1° latitude and longitude for the area 25°N - 70°N and 25°W - 40°E.

The daily measurement data of the DWD and the LfU are first de-trended (using the trend of a linear regression) to eliminate the influence of climate change, which is already evident in the 10-year data, and to eliminate potential biases at the beginning and end of the time series. Then daily anomalies are calculated using the 31-day moving long-term (2010–2019) averages of each calendar day. The anomalies now make it possible to assess whether the individual days deviate particularly strongly from the normal state, whether, for example, particularly warm or cold days or particularly high or low air pressure prevailed.

**Figure 1:**
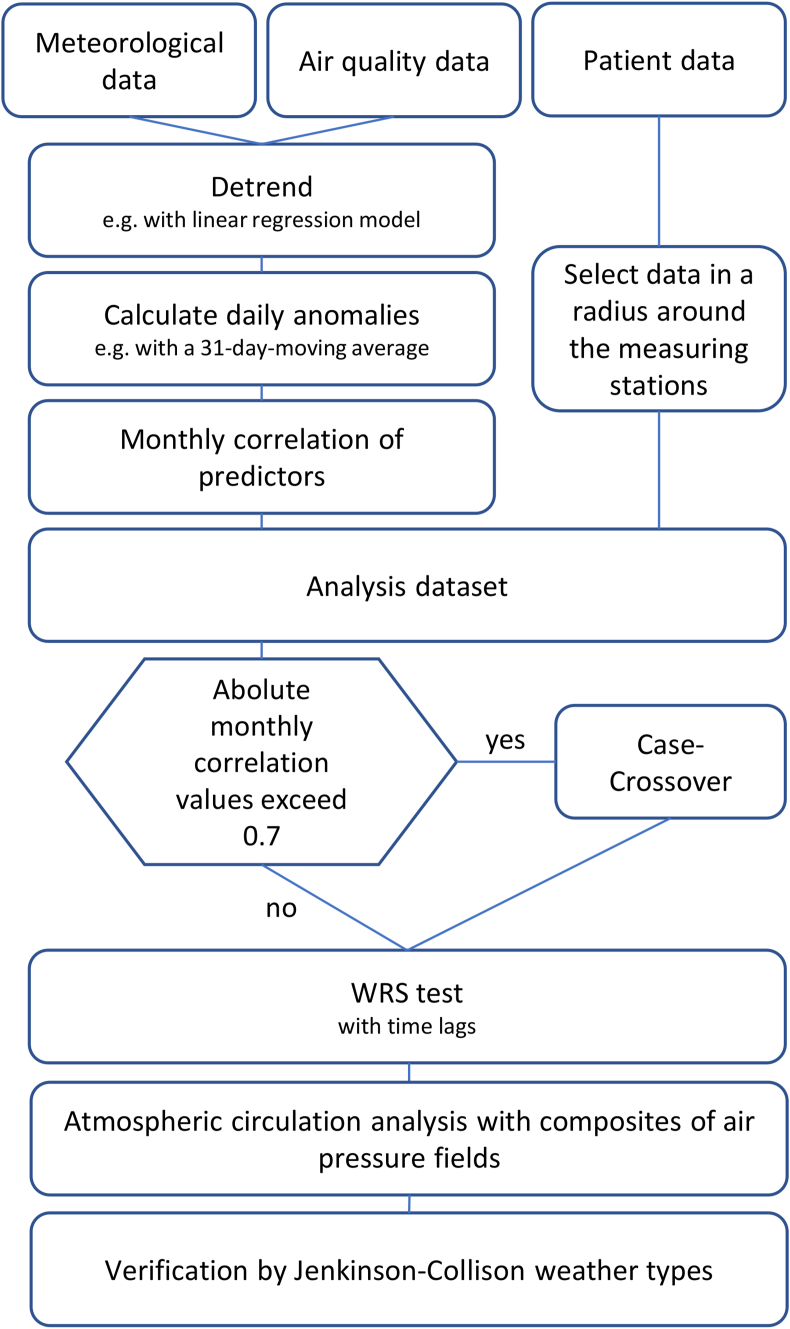
Flowchart of the methods used in this study.

In the following statistical analyses only patients are considered who live within a maximum distance of 20 km from the measuring sites of the DWD. This selection of the patient data is necessary because weather and air pollutants are local phenomena and therefore have a limited range of effects. The radius of 20 km is the result of a compromise: on the one hand, as many patients as possible should still be included in the survey, and on the other hand, it must be ensured that they are still exposed to the influence of the measured weather and air pollutants. The method we developed for analyzing rAA is shown in Figure 1.

### 2.2 Case-crossover in preparation for the subsequent test procedure

To determine if weather and/or air pollutants have an impact on the occurrence of rAA, we must first determine which days should be included as rAA-influencing and rAA-independent in the subsequent testing methodology. Since we want to examine not only the conditions on the day of the rAA itself, but also the preceding atmospheric conditions, the 13 days preceding an rAA were also included in the sample of rAA-influencing days.

Since it is known that the meteorological variables and the air pollutants can be interdependent (for example, the ozone concentration depends strongly on air temperature), it must be ensured that these influences are minimized in the selection of the samples. We have developed our own case-crossover procedure for this purpose, based on the approaches of Pinheiro et al. (2014) and Olschewski et al. (2021):

We first check whether the meteorological and air quality data under study are correlated (with a Spearman correlation (Spearman, 1904), individually for each month of the year). Variables that are strongly correlated (|r| > 0.7) with each other and do not belong to the same group (weather or air pollutants) must be subjected to a case-crossover procedure. This means that, for example, daily minimum temperature and daily mean temperature, which are usually strongly correlated, are not tested. However, air temperature and ozone, if strongly correlated, must go through the case-crossover process.

This case-crossover is performed with the strongly correlated variables in the corresponding months. To be able to exclude an influence of variable B on variable A, values as similar as possible are sought for variable B in the same month over the entire study period for all rAA days, including the preceding 13 days. Thus, variable B contains comparable values in both the case and control periods. The associated values of variable A in the most appropriate control period are then considered independent of variable B and can be used in the subsequent test procedure. The similarity of the two-week case and control periods is determined by the sum of the absolute differences of the anomalies. The differences are additionally weighted in the form of a half Gaussian curve, which assigns a higher priority to the days that are closer to the rAA case day.

### 2.3 Wilcoxon rank-sum test (WRS)

WRS, also known as U-test (Mann and Whitney, 1947; Wilcoxon, 1945), is conducted to find relationships between meteorological and air quality conditions and the occurrence of rAA. Time lags of up to two weeks are also considered to identify any lagged influence of weather and air pollutants on rAA.

With the help of the WRS test, it can be checked whether the meteorological and air-hygienic variables on days (including a preceding period) with rAA differ significantly from periods without rAA. The analyses should have the highest temporal resolution possible to reveal seasonal relationships. The choice of temporal resolution depends on the amount of patient data available. In our case, a single-month analysis would have resulted in sample sizes that were too small, so we consider the rAA of three consecutive months together and then shift our analysis period by one month each time. Thus, WRS tests are performed for January to March, then February to April, March to May, and so on. In this way, we obtain a more realistic representation of the seasonal course that is not limited to predefined seasonal sections.

Depending on the variables and month under consideration, a case-crossover analysis is applied beforehand. If case-crossover is necessary, which was determined via the correlation of the individual variables, the samples consist of the days on which rAA occurred together with the 13 days before, and the selected control sequences defined by the case-crossover methodology. If no case-crossover is necessary, the samples consist of the days on which rAA occurred together with the 13 days before, and all other days in the corresponding months that are not within the influence of an rAA.

### 2.4 Composites of air pressure fields: atmospheric circulation analysis

In order to further analyze the results of the WRS in the context of the underlying meteorology, so-called air pressure composites are calculated using the ERA5 reanalysis data. First, mean values are calculated from the air pressure fields for the corresponding months that emerged as relevant from the WRS to determine the representative climatological condition. Next, those days are singled out that correspond to significant anomaly results before or during an rAA - for example, days with particularly high winter air temperatures before rAAs occurred. The air pressure fields on these days are also averaged and the mean state of the atmosphere prevailing during the days important for rAA is obtained. The difference between the climatology composite and the anomaly composite is formed, yielding a picture that shows the differences in atmospheric air pressure between rAA-relevant and non-relevant days.

### 2.5 Jenkinson-Collison (JC) weather types

In order to further verify the statements of the composite analysis, daily Jenkinson-Collison (JC) weather types (Jenkinson and Collison, 1977) were calculated using air pressure data from the ERA5 reanalysis for Augsburg and Munich. The JC method was developed to objectively capture the 27 atmospheric weather types (WT) that exist in the Lamb weather type system. They include eight purely advective (or directional) types based on wind direction (e.g., SE or southeast); A and C types, representing anticyclonic or cyclonic pressure patterns, respectively, but no coherent flow direction; 16 hybrid types, representing directional types with either anticyclonic isobaric curvature (e.g. AN or anticyclonic northerly direction) or cyclonic curvature (e.g. CSE or cyclonic southeasterly direction); and type U (indifferent), representing patterns with weak pressure gradients such that neither flow direction nor vorticity can be identified.

## 3 Theory

The suggestion that weather may have an influence on aortic ruptures has been investigated for many years. However, all publications to date on this topic show a very wide range of results. The most studied variables in relation to rAA are atmospheric pressure and air temperature.

Two recent meta-studies (Choong et al., 2019; Wu et al., 2018) on the previous published analysis results show that arguably rAA are somewhat more frequent in the fall and winter months, but a significant relationship between rAA and weather does not appear to exist universally. The authors note that the large heterogeneity in sample size, the selected methodological approach, and the consideration of seasonality, among other factors, do not yet allow generalized conclusions about the association of meteorology and rAA.

Individual studies very well come to significant results, such as that rAA are related to low air pressure (Bown et al., 2003; Harkin et al., 2005; Jamshidi and MacKenzie, 2011; Killeen et al., 2008; Kurtoglu et al., 2004; Smith et al., 2008) or to low air temperatures (Basu et al., 2012; Brightwell et al., 2014; Mestres et al., 2020). However, there are also numerous studies that cannot confirm these relationships (Ishikawa et al., 2012; Molacek et al., 2013; Sterpetti et al., 1995; Urbanek et al., 2015; Verberkmoes et al., 2012) or even provide contrary results (such as a significant association of rAA with high atmospheric pressure (Kacanski et al., 2019)). Analyses with sunshine duration and the dependence of rAA on lunar phases have also been undertaken, but do not reach consistent results either (Ishikawa et al., 2012; Kózka et al., 2014; Takagi et al., 2017).

The pathophysiological mechanisms underlying the effect of meteorological influences on rAA remain equally uncertain. Although it is hypothesized that increased stress on an existing aneurysm may be caused by a reduction in extraluminal pressure (as a result of lower atmospheric pressure) to increase the pressure differential across the aortic wall, thereby increasing the risk of rupture (Choong et al., 2019). In addition, low atmospheric pressure has been reported to decrease oxygenation of arterial blood, which activates endocrine and autonomic responses leading to an increase in blood pressure levels (Kaminski et al., 2016). But often there is a lack of robust data on the patient's condition during rAA, whether due to a simple lack of appropriate databases or the falsification of measurement results through the administration of drugs by emergency physicians before patients arrive at the hospital.

The studies to date on the influence of weather and air pollutants on rAA are methodologically very heterogeneous. The number of patients, the number of years for which data are available, the size of the study area, how many atmospheric or air-hygienic parameters and, if applicable, a combined influence were investigated, and the statistical methods used differ greatly.

In the literature review, it is noticeable that no research has yet been done to determine whether the meteorological data show a trend in their study period. A climatic trend in the data can possibly lead to biases at the beginning and end of the time series. In many studies, it is also not apparent whether the patients were under the influence of the meteorological data used at the time of rupture. Exceptions to this are, for example, the studies by Brightwell et al. (2014), Robert et al. (2014), and Mestres et al. (2020), which ensure that patient residences and location of monitoring stations are matched.

The most commonly used statistical methods to date to describe the characteristics of rAA and associations with weather are chi-square tests, Student t tests, the nonparametric U test, and linear and multiple logistic regressions (Schuld et al., 2013). We are not aware of any study that attempts to go beyond the results of the univariate and multivariate tests to explore the underlying climatological relationships.

## 4 Results

In the following chapter, the results of our methodology are explained using the data available to us from southern Germany.

### 4.1 Patient data structure

**Figure 2:**
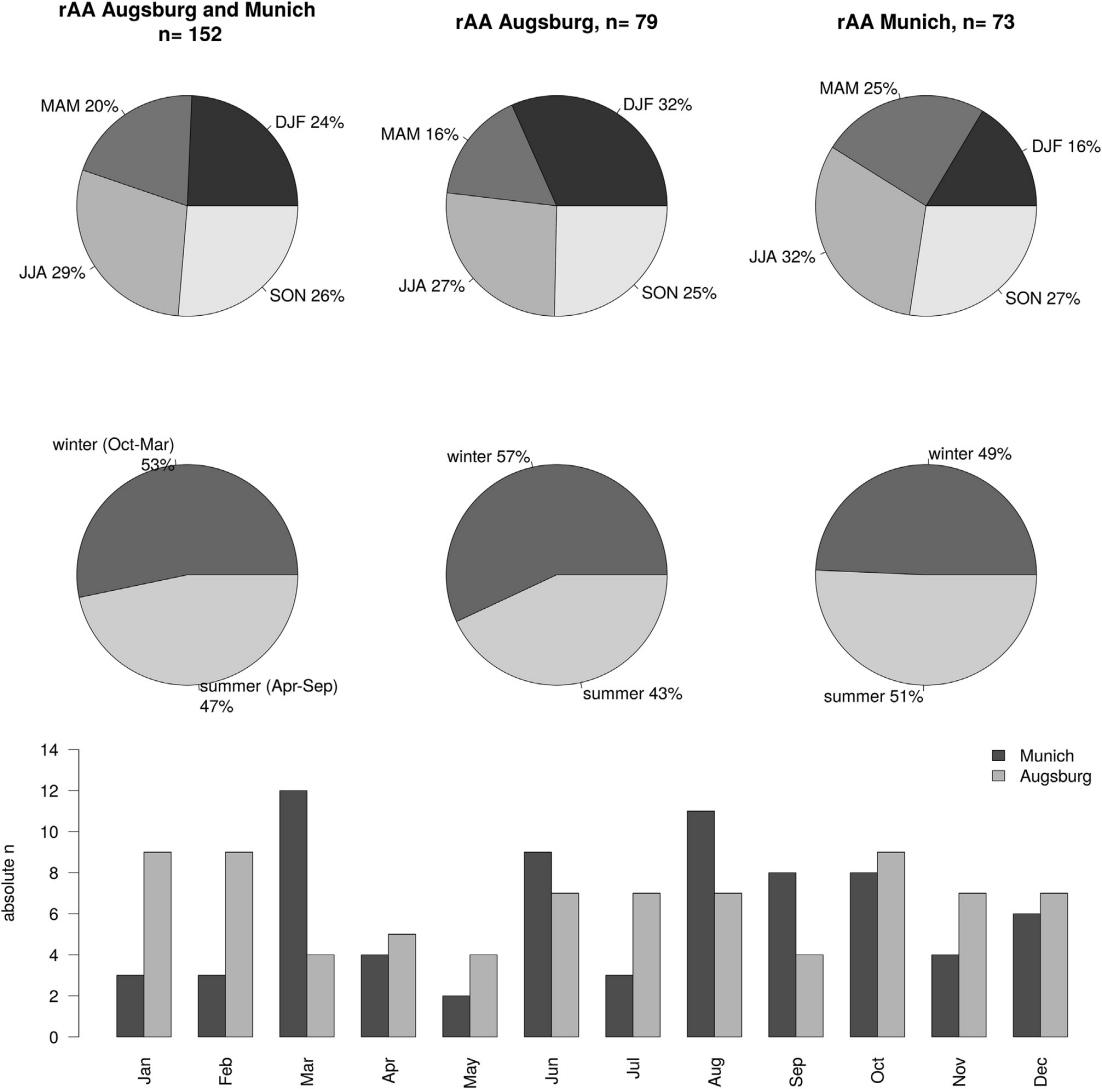
Absolute monthly (bottom) and relative seasonal und half-year (pie charts) frequencies of rAA in Augsburg and Munich from 2010-2019 (note that only patients with their residence within 20 km around the DWD measurement stations are considered).

In the investigated period from 2010-2019, 140 patients with rAA were admitted to the University hospital of Augsburg and 148 patients to the University hospital rechts der Isar in Munich. After narrowing down the patient data to a radius of 20 km around the DWD stations, 79 cases remain in Augsburg and 73 in Munich (see Figure 2). In Augsburg, most rAA occurred in October, February, and January during 2010–2019, and the fewest cases occurred in March, May, and September. In Munich, the most cases occurred in March, August, and June, and the fewest rAA cases are found in May, January, and February. In Augsburg, slightly more cases occur in the winter half-year (October–March, 57%) than in the summer (43%), whereas in Munich, rAA are more evenly distributed over the winter (49%) and summer half-years (51%). The frequency of rAA from year to year is highly variable and also shows no systematics between the two cities studied.

### 4.2 Results of case-crossover WRS

Since it can be assumed that the study regions, which are relatively close in space (the distance between the two cities is about 50 km), should be subject to similar exposure-outcome mechanisms between weather/air constituents and rAA, the following analyses, based on the results of the WRS, focus on the similarities between the two cities.

#### 4.2.1 Results of the case-crossover procedure

The single-month correlation analyses between all meteorological variables and the air constituents in the case-crossover study have shown significant results (|r| > 0.7) for several combinations of variables. Therefore, for these variables, a case-crossover procedure was applied for the selection of the samples to be included in the WRS in the corresponding months. Thus, especially for the combination of air temperature and ozone in the summer months and for wind speed, ozone and nitrogen oxides, respectively, in the winter months, it became necessary to apply case-crossover. This can ensure that the samples used in the WRS tests are not perturbed by meteorological-air content correlations.

#### 4.2.2 Results of the WRS tests

**Figure 3:**
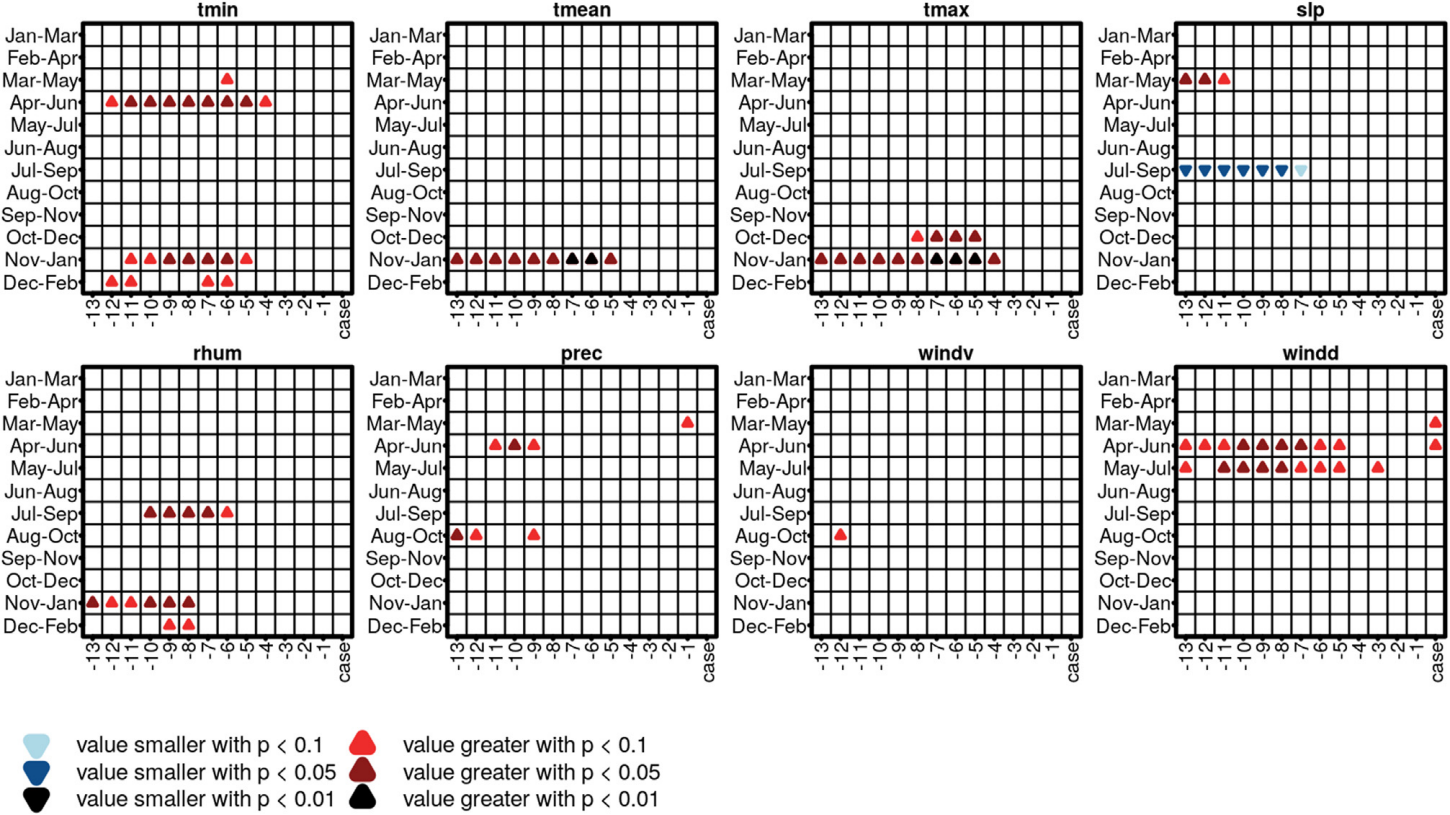
Results of the WRS for the meteorological variables tmin (daily minimum air temperature), tmean (daily mean air temperature), tmax (daily maximum air temperature), slp (daily mean sea level pressure), rhum (daily mean relative humidity), prec (daily precipitation sum), windv (daily mean wind velocity) and windd (daily mean wind direction). Shown are only results that have statistically significant values (p < 0.1, p < 0.05 and p < 0.01) in both cities studied. Upward pointing triangles indicate that the value before or on the day of the rAA were significantly higher than average, downward pointing triangles indicate lower than average values. "case" indicates the day of the rAA, to the left are the preceding days.

Because aortic ruptures from three consecutive months (of running three-month combinations within the year) are included in a WRS test, each sample contains a different number of cases. For the WRS tests containing only the days when rAA occurred (column “case” in Figure 3), the following sample sizes result for the rAA days (Augsburg/Munich, respectively): Jan–Mar 22/18, Feb–Apr 18/19, Mar–May 13/18, Apr–Jun 16/15, May–Jul 18/14, Jun–Aug 21/23 Jul–Sep 18/22, Aug–Oct 20/27, Sep–Nov 20/20, Oct–Dec 23/18, Nov–Jan 23/13, and Dec–Feb 25/12. With the addition of the first lag day, the sample sizes double, and with each additional lag day, the quantities of the original "case" sample sizes add up. This means that the results of the WRS tests which contain only the day of the rAA have only a few cases, but sample sizes increase rapidly with the addition of lag days and thus the test also gains robustness.

Figure 3 shows the results of the WRS tests. Here, we do not show the individual results for the two cities, but only those results that occur simultaneously for both cities and have a significance level of at least p < 0.1, p < 0.05, and p < 0.01. There are significant differences in the meteorological variables with and without rAA for both cities studied. These significant differences between periods with and without rAA occurred can be grouped into variable combinations:

• In the months of April through June the days preceding an rAA are characterized by elevated daily minimum temperatures, above-average precipitation, and higher (i.e., more westerly) wind flow.
• In mid and late summer (July–September) the days before an rAA are characterized by below-average air pressure and increased relative humidity.
• In autumn and winter (October to January) higher than usual daily minimum, mean and maximum temperatures before rAA occurred.

It is also striking that the significant differences mostly exist up to two weeks before the rupture occurred. The results of the WRS on air pollutants show basically no similarities for the two cities studied.

### 4.3 Typical weather conditions that have an influence on the occurrence of aortic ruptures in southern Germany

The three groups of variables identified by the WRS are now examined in more detail for their atmospheric circulation background. In the DWD data, the days before and during rAA are selected which correspond to the conditions of the variable groups. For these days a composite of the ERA5 air pressure data is formed and compared with the air pressure fields independent of rAA.

#### 4.3.1 Rainy northwest-type in spring

**Figure 4:**
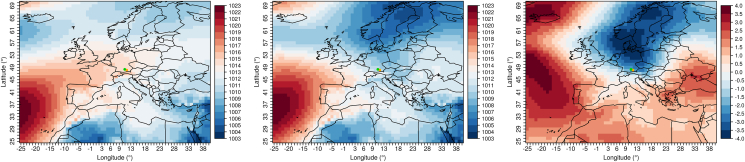
Composites of the ERA5 air pressure fields. Left: Climatology of the months April to June (2010–2019). Center: Mean of all days meeting the conditions above average rainfall, above daily minimum temperature, and an above average wind speed within twelve days before an rAA (also see Section 4.3.1). Right: Difference of the left two plots. Values in hPa. The dots mark the two investigated cities Augsburg (green) and Munich (orange).

For the months of April through June and a maximum of twelve lag days before an rAA, days are selected that have above average rainfall, daily minimum temperature, and an above normal wind speed. 87% (27 out of 31 rAA) of all rAA from April to June of the years 2010–2019 are connected with these conditions. The air pressure fields matching these days from the ERA5 reanalysis data are now averaged and compared to the air pressure field shown by the April through June climatology (see Figure 4). The shift of the Azores High to the north and a pronounced low-pressure area over Scandinavia can be seen, which leads to abundant precipitation over our study area. This results in contrary cloudy weather with showers (so-called "April weather"). The elevated daily minimum temperatures can be explained by the fact that the prevailing cloud cover at night prevents the emittance of thermal radiation and thus a cooling of the air near the ground.

#### 4.3.2 Humid weather in summer

**Figure 5:**
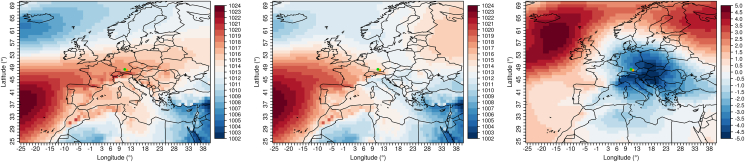
Composites of the ERA5 air pressure fields. Left: Climatology of the months July to September (2010–2019). Center: Mean of all days meeting the conditions below average atmospheric pressure and above average relative humidity within 13 days before an rAA (also see Section 4.3.2). Right: Difference of the left two plots. Values in hPa. The dots mark the two investigated cities Augsburg (green) and Munich (orange).

For the months of July through September and a maximum of 13 lag days, days are selected that have below average atmospheric pressure and higher than normal relative humidity. 90% (36 out of 40 rAA) of all rAA from July to September are associated with these conditions. The corresponding air pressure fields in Figure 5 show higher air pressure over northern Europe (blocking effect), and lower air pressure over the Mediterranean region. This allows air from an easterly direction to flow into Central Europe, connected with contrary, humid, and thundery weather conditions.

#### 4.3.3 Warm southwest weather in autumn and winter

**Figure 6:**
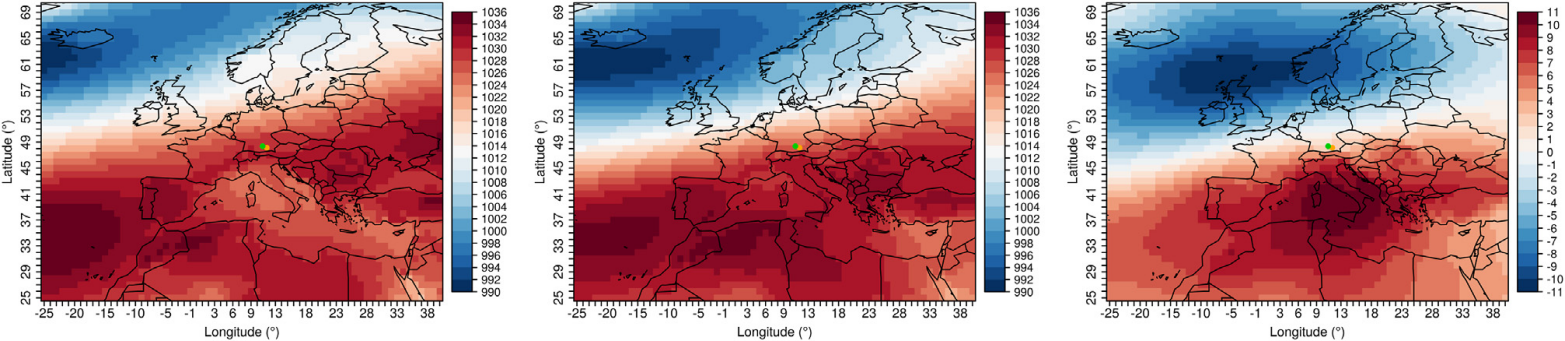
Composites of the ERA5 air pressure fields. Left: Climatology of the months October to January (2010–2019). Center: Mean of all days meeting the conditions above average minimum, mean and also maximum daily air temperatures within eleven days before an rAA (also see Section 4.3.3). Right: Difference of the left two plots. Values in hPa. The dots mark the two investigated cities Augsburg (green) and Munich (orange).

For the months of October through January and a maximum of eleven lag days, days are extracted that have above average minimum, mean and maximum daily air temperatures. 68% (36 out of 53 rAA) of all rAA from October to January are related with these conditions. The composite images in Figure 6 show that on these days, which are important for the occurrence of rAA, a weather type with south to southwest air flow is established, which can be attributed to higher air pressure south of the Alps and lower pressure in northern Europe. This allows the inflow of relatively warm air to our target region, which may also be accompanied by the local weather phenomenon of alpine foehn.

### 4.4 JC WTs of the composites

**Figure 7:**
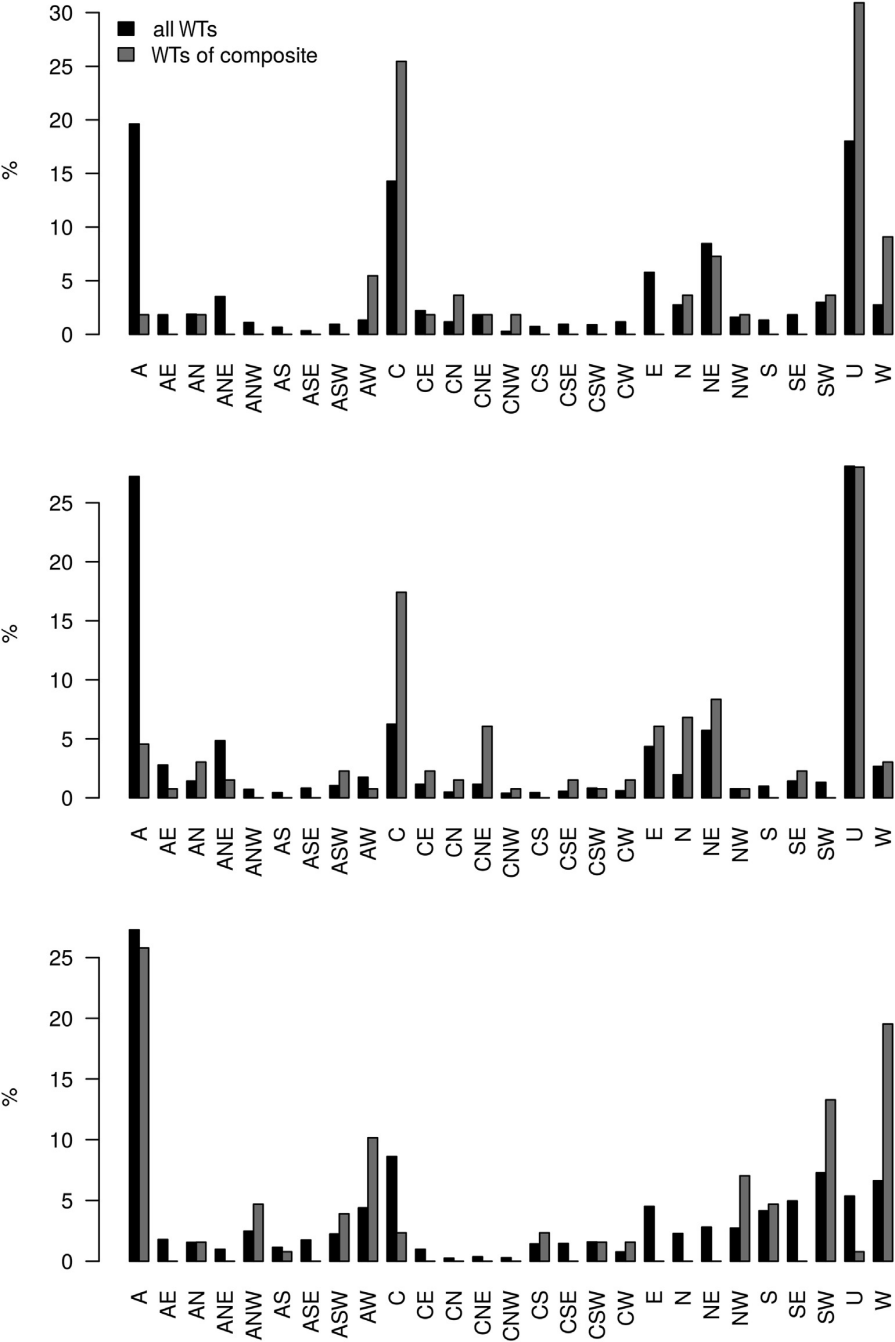
Percentage occurrence of Jenkinson-Collison weather types (WT) for Augsburg and Munich for 2010–2019. Top: For all WTs from April to June (black signature) and the days meeting conditions from Section 4.3.1 (Rainy northwest-type in spring, grey signature). Center: For all WTs from July to September and the days meeting conditions from Section 4.3.2 (Humid weather in summer). Bottom: For all WTs from October to January and the days meeting conditions from Section 4.3.3 (Warm southwest weather in autumn and winter). A = anticyclonic, C = cyclonic, S = South, W = West, N = North, E = East, U = indifferent weather type.

Figure 7 shows the percent occurrence of JC weather types in Augsburg and Munich, matching the composites from the previous section. On the one hand, all WTs occurring for the relevant months are shown and alongside the weather situations that occurred on days identified by the composites which affect rAA.

The top row of Figure 7 shows the WTs for the *rainy northwest-type in spring*. While anticyclonic (A), cyclonic (C), and the indifferent weather type (U) occur most frequently averaged over the months of April through June of 2010–2019, the frequencies of occurrence of weather types on rAA-inducing days change significantly. Almost all anticyclonic weather situations, i.e. determined by high pressure, decrease significantly, while cyclonic (C, CN, CNW) and also westerly weather conditions (AW, CNW, NW, W) occur much more frequently in comparison. This frequency distribution of the weather situations consequently underlines the picture that has already emerged from the composite analysis.

Likewise, the second rAA-inducing weather pattern, *humid weather in summer*, is associated with a strong decrease in anticyclonic weather types compared to the long-term average (see Figure 7, middle row). The increase of cyclonic as well as northerly and easterly inflow directions (C, CNE, E, N, NE) proves the theory that on these days contrary summer weather with strong influence of air masses from easterly direction is to be expected in southern Germany.

The third identified weather type affecting aortic ruptures in autumn and winter, namely *warm southwest weather*, is characterized by a strong increase of west and south WTs (ANW, ASW, AW, CS, CW, SW, W) when considering JC weather patterns, while mainly pure cyclonic (C), and north and east WTs (E, N, NE, SE) become less important. So, also in this case, the assumptions about the prevailing weather of the days important for the rAA are verified.

## 5 Discussion

The accumulation of rAA in the winter half-year mentioned in the literature applies to only one of the cities we investigated (Augsburg). In Munich the two months with the highest number of ruptures were in summer. Also, the so far most mentioned claims that rAA occur more frequently at low air pressure and cold air temperatures and the related pathophysiological explanations cannot fully be confirmed by us. Only in the summer months a lower air pressure plays a significant role, for other months this is not the case. Moreover, the results of the WRS test show exclusively positive anomalies for the air temperatures. The underlying pathophysiological mechanisms that lead to the rupture of the aneurysm accompanied by the weather types we found need to be explored in further medical studies and are not the objective of our present work. Because we did not have complete data on the age and sex of the patients, it was not possible to analyze their influences on our study results.

When investigating meteorological and air-hygienic influences on the patients, it is essential that these people were actually under the influence of the assumed environmental parameters at the time of the aortic rupture. Therefore, on the one hand, we had to assume that the patients were at least in the vicinity of their place of residence on the day of their admission to the hospital. And on the other hand, we had to estimate in which radius around the measurement sites the meteorological and air-hygienic measurement data still retain their validity. Therefore, we tested different residential radii of 10, 20 and 30 km around the measurement sites and analyzed how this affects the results. Only minor deviations in the results were found and they did not affect the conclusions of our study. As a compromise between decreasing measurement site-actual patient exposure relationships with increasing spatial distance and the reduction of sample size with smaller analysis radius, we decided to use a radius of 20 km in the results shown here.

To further validate the results of our analyses, in addition to the WRS test, two other test procedures were used to check whether the frequency distributions of two different samples differ from each other. The Cramér-von Mises criterion and the Anderson-Darling test were used for this purpose. The two alternative test procedures underlined the results of the WRS test. Since the WRS test appears to be the most stringent of the three test procedures, our analysis results are based on its results.

In our study, meteorological conditions as well as air pollutants were investigated. The results show that in our case the meteorological influences are more important than the air pollutants. However, if the investigated cities are considered separately, there are quite significant anomalies in the air constituents, which should be investigated in more detail in further studies. Since we have attached importance to evaluate only the similarities of both cities, in order to make the results consistent and meaningful, we do not make any statements about the influence of air pollutants on aortic ruptures in this paper.

Furthermore, it should be noted that not all ruptures were associated with the weather conditions we mention, but that there are also several other conditions that can prevail. This can be seen for instance in the composite analysis for autumn and winter, where only just under 70 percent of the ruptures were accompanied by increased air temperatures.

The very different study results reported in the literature on rAA in relation to meteorological factors (mainly air pressure and air temperature) and also the fact that our results show strong seasonal differences suggest that the risk of rAA due to weather phenomena may vary considerably from region to region and from season to season. It is also possible that regional extreme weather events like the alpine foehn play a crucial role in the rupture of an aortic aneurysm.

We argue that the rAA-associated weather conditions identified in our study are not necessarily valid for other regions of the world. However, the developed methodology can be applied to other regions, and can advance the development of prediction and prevention strategies. This gains even more importance in the scope of climate change, where significant changes like increases of heat waves and a higher persistence of specific weather types occur in many parts of the world.

## 6 Conclusions

We have developed a methodology to test the influence of weather and air pollutants on rAA in a statistically correct and meaningful way. This allows for a very detailed seasonal analysis that also includes any period before the rupture occurred (in our case, two weeks). Important here is the careful preparation of the data, such as a possible removal of the climatic trend or the calculation of the daily anomalies. In addition, it must be ensured that only patients who were spatially exposed to the weather or air pollutant influence are considered. We solved this using the known residence of the patients and a radius of 20 km around the weather measuring stations.

A WRS test can then check whether days on or before an rAA differ from days without an rAA. However, it must first be checked whether the weather and air pollution data used influence each other and it must be ensured that only the relevant variables result in an influence on the result of the WRS. We solved this by a case-crossover procedure that checks the correlations of all variables and if a correlation is found, adjusts the samples to be used for the WRS test. Applying this test scheme to the rAA data from Augsburg and Munich have shown that at different times of the year specific anomaly groups occur in both southern German cities before rAA.

For the months of April through June and a maximum of twelve lag days before an rAA, days with above average rainfall, high daily minimum temperature, and a greater than normal wind direction are found. For the months of July through September and a maximum of 13 lag days before an rAA, days are found with below average atmospheric pressure and higher than normal relative humidity. For the months of October through January and a maximum of eleven lag days, days with above average minimum, mean and also maximum daily air temperatures precede an rAA.

In order to verify which atmospheric conditions and circulation dynamic backgrounds led to these significant anomalies before rAA, the matching air pressure fields over an extended European area were considered. These then reveal typical large-scale weather patterns that appear to have an influence on rAA in southern Germany.

## Declarations

### Author contribution statement

Irena Kaspar-Ott: Conceived and designed the experiments; Performed the experiments; Analyzed and interpreted the data; Contributed reagents, materials, analysis tools or data; Wrote the paper.

Patrick Olschewski: Conceived and designed the experiments; Contributed reagents, materials, analysis tools or data.

Stephanie Koller: Conceived and designed the experiments.

Alexander Hyhlik-Duerr, Elena Streck, Hans-Henning Eckstein & Oksana Radu: Contributed reagents, materials, analysis tools or data.

Elke Hertig: Conceived and designed the experiments; Analyzed and interpreted the data.

### Funding statement

This work was supported by Deutsche Forschungsgemeinschaft (408057478).

### Data availability statement

The authors do not have permission to share data.

### Declaration of interests statement

The authors declare no conflict of interest.

### Additional information

No additional information is available for this paper.
